# Quantifying lumbar paraspinal intramuscular fat: Accuracy and reliability of automated thresholding models

**DOI:** 10.1016/j.xnsj.2024.100313

**Published:** 2024-01-24

**Authors:** E.O. Wesselink, J.M. Elliott, A. Pool-Goudzwaard, M.W. Coppieters, P.P. Pevenage, A. Di Ieva, K.A. Weber II

**Affiliations:** a Faculty of Behavioural and Movement Sciences, Amsterdam Movement Sciences – Program Musculoskeletal Health, Vrije Universiteit Amsterdam, Amsterdam, The Netherlands; b Division of Pain Medicine, Department of Anesthesiology, Perioperative and Pain Medicine, Stanford University School of Medicine, Palo Alto, CA, United States; c The University of Sydney, Faculty of Medicine and Health and the Northern Sydney Local Health District, The Kolling Institute, Sydney, Australia; d SOMT University of Physiotherapy, Amersfoort, The Netherlands; e Menzies Health Institute Queensland, School of Health Sciences and Social Work, Griffith University, Brisbane and Gold Coast, Australia; f MRI Centrum, Amsterdam, The Netherlands; g Computational Neurosurgery (CNS) Lab, Macquarie Medical School, Faculty of Medicine, Health and Human Sciences, Macquarie University, Level 1, 75 Talavera Road, Sydney, NSW 2109, Australia

**Keywords:** Machine learning, Thresholding, Magnetic resonance imaging, Back muscles, Adiposity, Low back pain

## Abstract

**Background:**

The reported level of lumbar paraspinal intramuscular fat (IMF) in people with low back pain (LBP) varies considerably across studies using conventional T_1_- and T_2_-weighted magnetic resonance imaging (MRI) sequences. This may be due to the different thresholding models employed to quantify IMF. In this study we investigated the accuracy and reliability of established (two-component) and novel (three-component) thresholding models to measure lumbar paraspinal IMF from T_2_-weighted MRI.

**Methods:**

In this cross-sectional study, we included MRI scans from 30 people with LBP (50% female; mean (SD) age: 46.3 (15.0) years). Gaussian mixture modelling (GMM) and K-means clustering were used to quantify IMF bilaterally from the lumbar multifidus, erector spinae, and psoas major using two and three-component thresholding approaches (GMM_2C_; K-means_2C_; GMM_3C_; and K-means_3C_). Dixon fat-water MRI was used as the reference for IMF. Accuracy was measured using Bland-Altman analyses, and reliability was measured using ICC_3,1_. The mean absolute error between thresholding models was compared using repeated-measures ANOVA and post-hoc paired sample t-tests (α = 0.05).

**Results:**

We found poor reliability for K-means_2C_ (ICC_3,1_ ≤ 0.38), moderate to good reliability for K-means_3C_ (ICC_3,1_ ≥ 0.68), moderate reliability for GMM_2C_ (ICC_3,1_ ≥ 0.63) and good reliability for GMM_3C_ (ICC_3,1_ ≥ 0.77). The GMM (p < .001) and three-component models (p < .001) had smaller mean absolute errors than K-means and two-component models, respectively. None of the investigated models adequately quantified IMF for psoas major (ICC_3,1_ ≤ 0.01).

**Conclusions:**

The performance of automated thresholding models is strongly dependent on the choice of algorithms, number of components, and muscle assessed. Compared to Dixon MRI, the GMM performed better than K-means and three-component performed better than two-component models for quantifying lumbar multifidus and erector spinae IMF. None of the investigated models accurately quantified IMF for psoas major. Future research is needed to investigate the performance of thresholding models in a more heterogeneous clinical dataset and across different sites and vendors.

## Introduction

Low back pain (LBP) is a multifactorial condition [1] and the leading cause of disability worldwide [2] with a lifetime prevalence of 84% [3]. Although most LBP is considered self-limiting in nature, evidence suggests a substantial proportion of individuals develop recurrent or chronic symptoms leading to poor functional outcomes and a concomitant increase in health care costs [4]. While a number of different approaches to diagnose and manage LBP have been developed, the global burden of this disease continues to grow [5].

Magnetic resonance imaging (MRI) is frequently used during the clinical course of LBP to visualize presumed spinal pathology (eg, disc degeneration, modic changes, facet arthrosis) due to high soft tissue contrast [6]. One additional observation, but often not considered in the clinical work-up, is paraspinal intramuscular fat (IMF) [7]. Paraspinal IMF is hypothesized to be driven by a process of persistent or recurring LBP through ongoing effects of pain, inactivity, and inflammatory mechanisms [8]. In particular, IMF appears to occupy the space of atrophic muscle fibers leading to reduced muscle contractility [9], which could negatively impact day-to-day functioning.

The magnitude of lumbar paraspinal IMF reported in people with LBP varies considerably between studies [10], [11], [12]. While multiple factors may account for the variability in IMF across studies [13], determining its clinical relevance is further complicated by the impact of different methods used to assess IMF [14,15]. Dixon fat-water MRI is considered the current reference standard for quantifying IMF [16]. However, IMF is most commonly estimated by means of structural MRI sequences acquired routinely for LBP. These include conventional T_1_- and T_2_-weighted MRI [14] in which IMF can be quantified using different thresholding techniques [10,17,18]. This includes fully-automated thresholding models, which provide an advantage over semi-automated and manual thresholding models by being more objective and time-efficient [19]. Previous studies have shown high accuracy and reliability between automated and manual thresholding models [19], but the performance compared to Dixon MRI is yet to be more extensively investigated [20], highlighting the need for further research in this area.

Two commonly used fully-automated thresholding models for quantifying paraspinal IMF from MRI scans are Gaussian mixture modelling (GMM) [9,10] and K-means clustering [21,22]. Both of these models determine a single threshold to distinguishes between, and classify muscle from IMF (ie, a two-component model) [19] where voxels with intensities that are below or above the threshold are classified as muscle or IMF, respectively [23]. However, the performance of two-component thresholding models are challenged when the variance of the image histogram is not bimodally distributed (absence of clearly distinguishable muscle and IMF peaks in very lean or fatty muscles) [24] or when muscle is not clearly distinguishable from IMF due to partial volume effects (ie medium intensity voxels that contain similar proportions of muscle and IMF). However, three-component models used to distinguish fat and fibroglandular tissue with digital mammography [25] have shown to better fit the image histogram. It is plausible a three-component model could improve the accuracy and reliability of quantifying lumbar paraspinal IMF from conventional MRI.

Here we aim to compare the accuracy and reliability of automated thresholding models for the quantification IMF from the lumbar multifidus, erector spinae, and psoas major in patients with LBP. We used GMM and K-means models with two or three-components to quantify paraspinal IMF from T_2_-weighted MRI, and the performance of the automated thresholding models was assessed relative to a Dixon fat-water MRI reference. We hypothesized that three-component models would outperform two-component models and demonstrate higher accuracy and reliability.

## Material and methods

### Participants

Lumbar T_2_-weighted and Dixon fat-water MRI scans were retrospectively collected from 1 MRI center from 30 patients who were referred for medical diagnostic research due to their LBP (50% female; mean (SD) age: 46.3 (15.0) years). All personal identifying information was removed before the data were accessed. As imaging was not performed for the purpose of this study, we retrieved no additional clinical data. The study was approved by the institutional ethical committee of the Vrije Universiteit Amsterdam (reference number VCWE-2023-126R1).

### Image acquisition and processing

Imaging was performed on a 3.0 Tesla MR Scanner (Magnetom Vida, Siemens Healthcare, Erlangen, Germany). Axial Dixon fat-water images of the lumbar spine were acquired with a spin-echo sequence (TR: 3.0 ms, TE_1_: 12.0 ms, TE_2_: 23.67 ms, number of averages: 2, slice thickness: 4 mm, in-plane resolution: 0.94 × 0.94 mm, matrix size: 256 × 208, flip angle: 122°, pixel bandwidth: 558 Hz). Axial T_2_-weighted images were acquired with a spin-echo sequence (TR: 3.3 ms, TE: 9.4 ms, slice thickness: 3 mm, in-plane resolution: 0.59 × 0.59 mm, matrix size: 320 × 320, flip angle: 160°, pixel bandwidth: 256 Hz). Per institutional protocol, participants were placed supine with their hips and knees slightly bent (≈30°). One blinded rater manually segmented the muscles of interest (ie, left and right lumbar multifidus, erector spinae, and psoas major) slice-wice between the mid-vertebral level of L4 and upper endplate of S1 using anatomical cross-references as previously described [14].

### Thresholding models

The mathematical expressions for GMM and K-means are presented in the Supplementary Materials. We chose initialized methods as recommended for optimal thresholding performance and initialized the 50 times to correct for the stochasticity of initializing model parameters [26]. Both models (GMM, covariance type: ‘full’, initialization method: ‘kmeans’, max iterations: 1000, tolerance: 0.001 [27], and K-means, initialization method: ‘k-means++’, max iterations: 1,000 and tolerance: 0.001) [27] were performed using the ScikitLearn (0.241) library. The thresholds for classifying muscle from IMF were determined separately for each muscle using the manual muscle segmentations. In total, 4 thresholding models were compared using two-components (GMM_2C_ and K-means_2C_) and novel three-components (GMM_3C_ and K-means_3C_).

### IMF calculations

IMF was calculated per muscle, separately left and right for the lumbar multifidus, erector spinae, and psoas major. For the Dixon fat-water MRI, we extracted the average fat signal and average water signal within each muscle using the muscle segmentations, and then calculated IMF as the percent of the total signal (fat + water) attributed to fat, by the following formula:

$$IMF_{Dixon}=\;\frac{\text{Signa}l_{\text{Fat}}}{\text{Signa}l_{\text{Fat}}+\text{Signa}l_{\text{Water}}}\;\times \;100$$

For the T_2_-weighted axial images, IMF was calculated as the percent of total volume (number of voxels) attributed to fat, by the following formula:

$$IMF_{T_{2}}=\;\frac{\text{Volum}e_{\text{Fat}}}{\text{Volum}e_{\text{Fat}}+\text{Volum}e_{\text{Muscle}}}\;\times \;100$$

For three-component analysis, the voxels corresponding to ‘undefined’ were assigned to fat, as we hypothesize that these voxels contain substantial fat concentrations due to partial volume effects, but are not clearly visible as fat depots [28].

### Statistical analyses

Baseline descriptive statistics were summarized separately per thresholding model (GMM_2C_; K-means_2C_; GMM_3C_ and K-means_3C_) and the reference standard (Dixon MRI). We assessed the accuracy and reliability of the thresholding models relative to the Dixon reference using ICC_3,1_, Bland-Altman analyses (average bias, standard deviation, and limits of agreement) and correlation plots. For the ICC_3,1_ values, <0.50 indicated poor reliability, 0.50 ≤ ICC_3,1_ < 0.75 indicated moderate reliability, 0.75 ≤ ICC_3,1_ < 0.90 indicated good reliability and ICC_3,1_ ≥ 0.90 indicated excellent reliability [29]. To assess differences in accuracy (using the mean absolute error) between the thresholding models relative to the Dixon reference, we used repeated-measures ANOVA with main effects for algorithm (ie, GMM and K-means) and component approach (ie, two and three-component) and an algorithm by component interaction term. Residuals between models were tested for normality and sphericity by the Shapiro–Wilk, Q–Q plots, and Mauchly's test of Sphericity. Post-hoc paired sample t-tests were used to compare the model with the lowest mean absolute error to the other models. All statistical analyses were performed using R-studio (Version 4.2.2) with an α < 0.05 as the threshold for statistical significance.

## Results

**Fig. 1:**
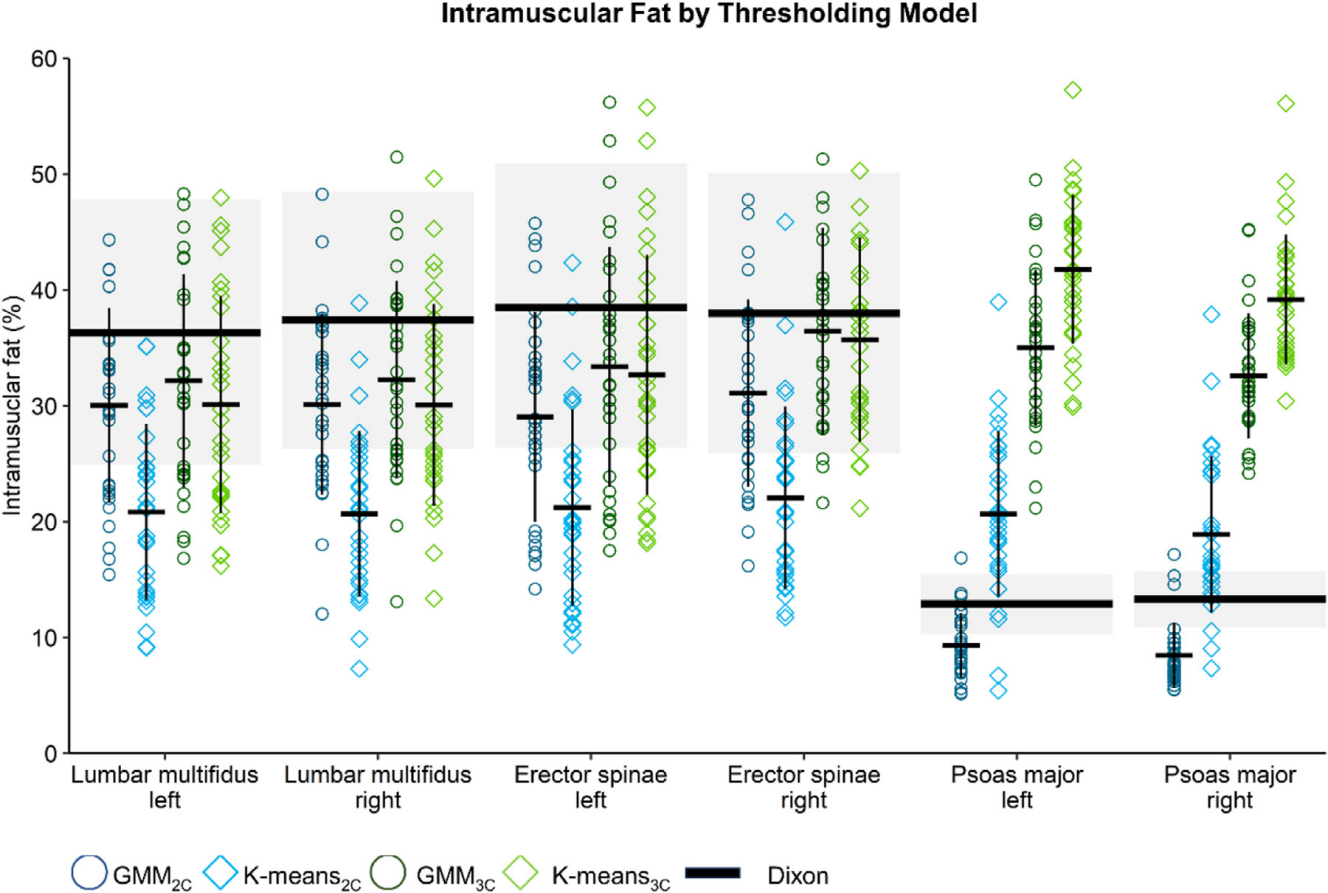
Mean percentage and error bars (1SD) of intramuscular fat for the 4 thresholding models. The reference of Dixon (black solid line) is presented with 1SD error bands (upper and lower) for each muscle.

Descriptive statistics for IMF across the four thresholding models (GMM_2C_; K-means_2C_; GMM_3C_; and K-means_3C_) are presented in Fig. 1 and Supplementary Table 1.

### Accuracy and reliability of two-component models: GMM_2C_ and K-means_2C_

**Fig. 2:**
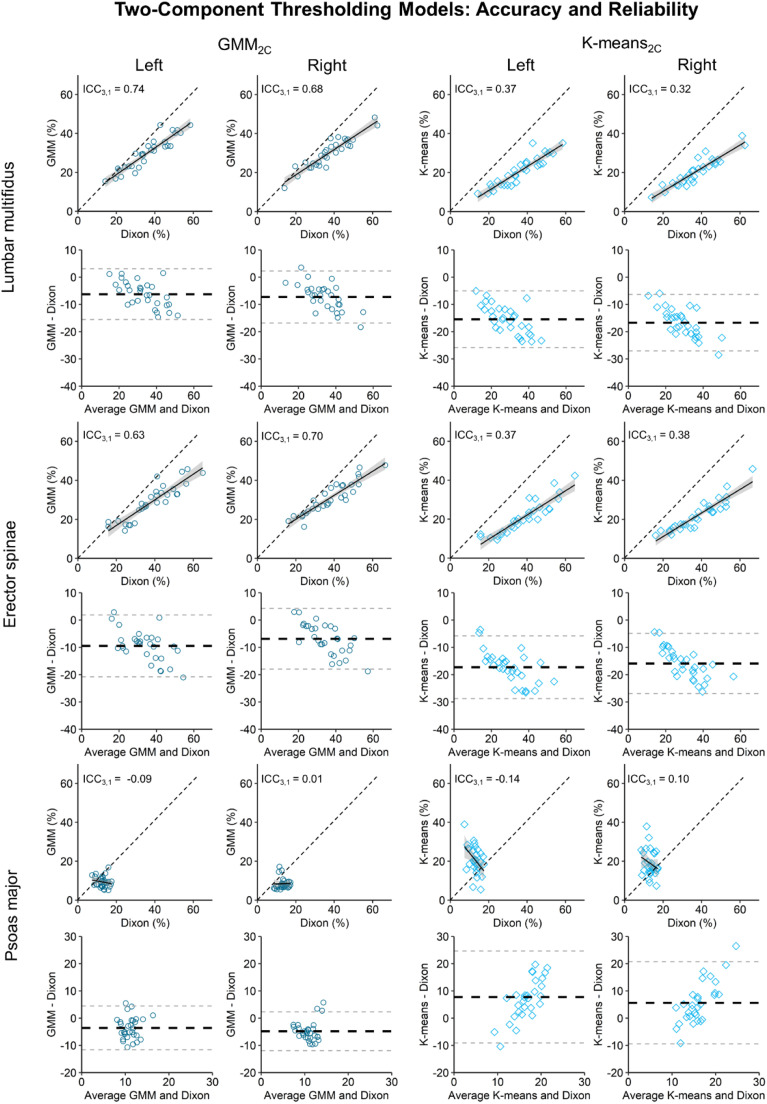
Performance of thresholding models using a two-component approach relative to the Dixon reference, using Bland-Altman and correlation plots. Dotted diagonal lines reflect perfect reliability (ICC_3,1_ = 1). Solid black line represents the trendline of the correlation plot with grey error bands (1SE).

Bland-Altman analyses showed that GMM_2C_ underestimated IMF for all muscles (average bias: −3.6% to −9.5%). K-means_2C_ underestimated IMF for the lumbar multifidus and erector spinae (average bias: −15.4% to −17.3%) and overestimated IMF for the psoas major (average bias: 5.6%–7.8%) (Table 1; Fig. 2). Reliability analyses revealed moderate reliability for the GMM_2C_ (ICC_3,1_ ≥ 0.63) and poor reliability for K-means_2C_ (ICC_3,1_ ≤ 0.38) for the lumbar multifidus and erector spinae. For the psoas major, poor reliability was found for both models (ICC_3,1_ ≤ 0.01) (Fig. 2).

**Table 1:** Performance of the two-component (Panel A) and three-component (Panel B) thresholding models versus Dixon MRI.

A.	GMM_2C_					K-means_2C_				
	Bland-Altman analysis				ICC_3,1_	Bland-Altman analysis				ICC_3,1_
	Bias	SD	LOA lower	LOA upper		Bias	SD	LOA lower	LOA upper	
LML	−6.2	4.8	−15.6	3.1	0.74	−15.4	5.3	−25.8	−5.1	0.37
LMR	−7.3	4.9	−16.8	2.3	0.68	−16.7	5.3	−27.1	−6.3	0.32
ESL	−9.5	5.8	−20.8	1.9	0.63	−17.3	5.9	−28.8	−5.8	0.37
ESR	−6.9	5.7	−18.0	4.2	0.70	−15.9	5.6	−26.9	−4.9	0.38
PML	−3.6	4.1	−11.6	4.5	−0.09	7.8	8.6	−9.1	24.6	−0.14
PMR	−4.8	3.6	−11.9	2.3	0.01	5.6	7.7	−9.4	20.7	−0.10

B.	GMM_3C_					K-means_3C_				
	Bland-Altman analysis				ICC_3,1_	Bland-Altman analysis				ICC_3,1_
	Bias	SD	LOA lower	LOA upper		Bias	SD	LOA lower	LOA upper	
LML	−4.1	4.8	−5.4	13.6	0.83	−6.2	5.4	−16.7	4.4	0.74
LMR	−5.1	5.0	−4.6	14.9	0.77	−7.3	5.4	−17.8	3.2	0.68
ESL	−5.1	5.3	−5.3	15.5	0.81	−5.8	5.7	−17.1	5.4	0.78
ESR	−1.5	5.1	−8.4	11.5	0.88	−2.3	6.1	−14.3	9.8	0.82
PML	22.1	7.5	7.4	36.8	-0.01	28.9	7.7	13.7	44.0	-0.01
PMR	19.3	5.6	8.3	30.3	0.01	25.9	6.4	13.3	38.4	-0.01

ESL, erector spinae left; ESR, erector spinae right; LML, lumbar multifidus left; LMR, lumbar multifidus right; LOA, limits of agreement; PML, Psoas major left; PMR, Psoas major right; SD, standard deviation.

### Accuracy and reliability of three-component models: GMM_3C_ and K-means_3C_

**Fig. 3:**
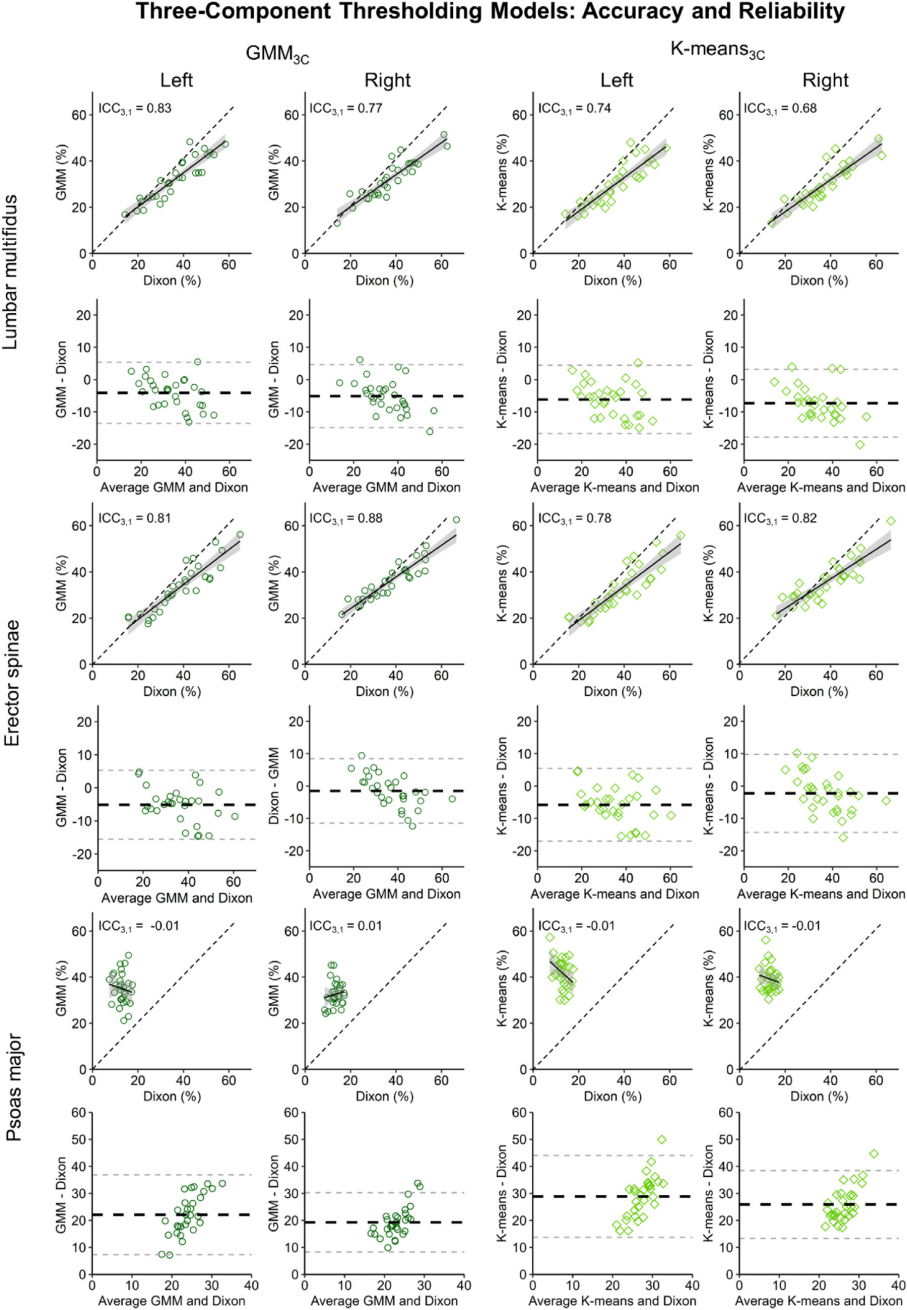
Performance of thresholding models using a three-component approach relative to the Dixon reference, using Bland-Altman and correlation plots. Dotted diagonal lines reflect perfect reliability (ICC_3,1_ = 1). Solid black line represents the trendline of the correlation plot with grey error bands (1SE).

Bland-Altman analyses showed that GMM_3C_ (average bias: −1.5% to −5.1%) and K-means_3C_ (average bias: −2.3% to −7.3%) underestimated IMF for the lumbar multifidus and erector spinae, and overestimated IMF for the psoas major (average bias: 19.3%–28.9%) (Table 1; Fig. 3). Reliability analyses revealed good reliability for GMM_3C_ (ICC_3,1_ ≥ 0.77) and moderate to good reliability for K-means_3C_ (ICC_3,1_ ≥ 0.68) for the lumbar multifidus and erector spinae. For the psoas major, poor reliability was found for both models (ICC_3,1_ ≤ 0.01) (Figure 3).

### Two-component versus three-component models

Repeated-measures ANOVA showed significant main effects (p < .001) for algorithm (ie, GMM or K-means) and components (ie, 2 or 3) for the mean absolute error of each muscle (normality and sphericity assumed, p > .05). A significant algorithm by component interaction was found for each muscle (p < .001), but not for the left psoas major (p = .203). Overall, GMM outperformed K-means for all muscles. The three-component models outperformed two-component models for the lumbar multifidus and erector spinae. However, two-component models outperformed three-component models for the psoas major. For the lumbar multifidus and erector spinae, the GMM_3C_ had the lowest mean absolute error, with the accuracy being significant compared to the other models (p ≤ .046). For the psoas major, the GMM_2C_ had the lowest mean absolute error compared to the other models, with the accuracy being significant compared to the other models (p ≤ .005), except for the right psoas major between the GMM_2C_ and K-means_2C_ (p = .346).

## Discussion

We investigated the accuracy and reliability of 4 automated thresholding models to measure paraspinal IMF from conventional T_2_-weighted imaging with respect to a Dixon fat-water reference. We found the accuracy and reliability of automated thresholding models are strongly dependent on the choice of algorithms, number of components, and the muscle to be assessed. Overall, the GMM performed better than K-means and three-component models performed better than two-component models for quantifying lumbar multifidus and erector spinae IMF. In particular, the GMM thresholding model with three-components (GMM_3C_) performed best with good accuracy and reliability. None of the investigated models adequately quantified IMF for the psoas major. The findings can guide the implementation of T_2_-weighted lumbar paraspinal IMF measures as secondary endpoints in clinical trials to further understand the role of muscle health in the persistence of LBP as well as treatment mechanisms.

The reported magnitude of lumbar paraspinal IMF in people with LBP varies between studies [10], [11], [12], and this may be due to the varying thresholding methods used from T_1_- or T_2_-weighted MRI [14,15]. For example, Shahidi et al. [10] reported substantially larger mean lumbar multifidus IMF values from T_2_-weighted MRI (IMF 45%–52% using GMM) than reported by Xiao et al. [22] (IMF 20.8%–30.4% using K-means) and Wesselink et al. [12] (IMF 22.0% using Otsu's thresholding). While multiple factors likely account for variability in IMF across these studies, including how (eg segmentation methods [15]) and where (eg unislice vs. multislice [13]) IMF was assessed as well as the sample characteristics (age, sex, body mass index, LBP status, etc. [30]), our findings highlight the impact of thresholding modelling choice.

**Fig. 4:**
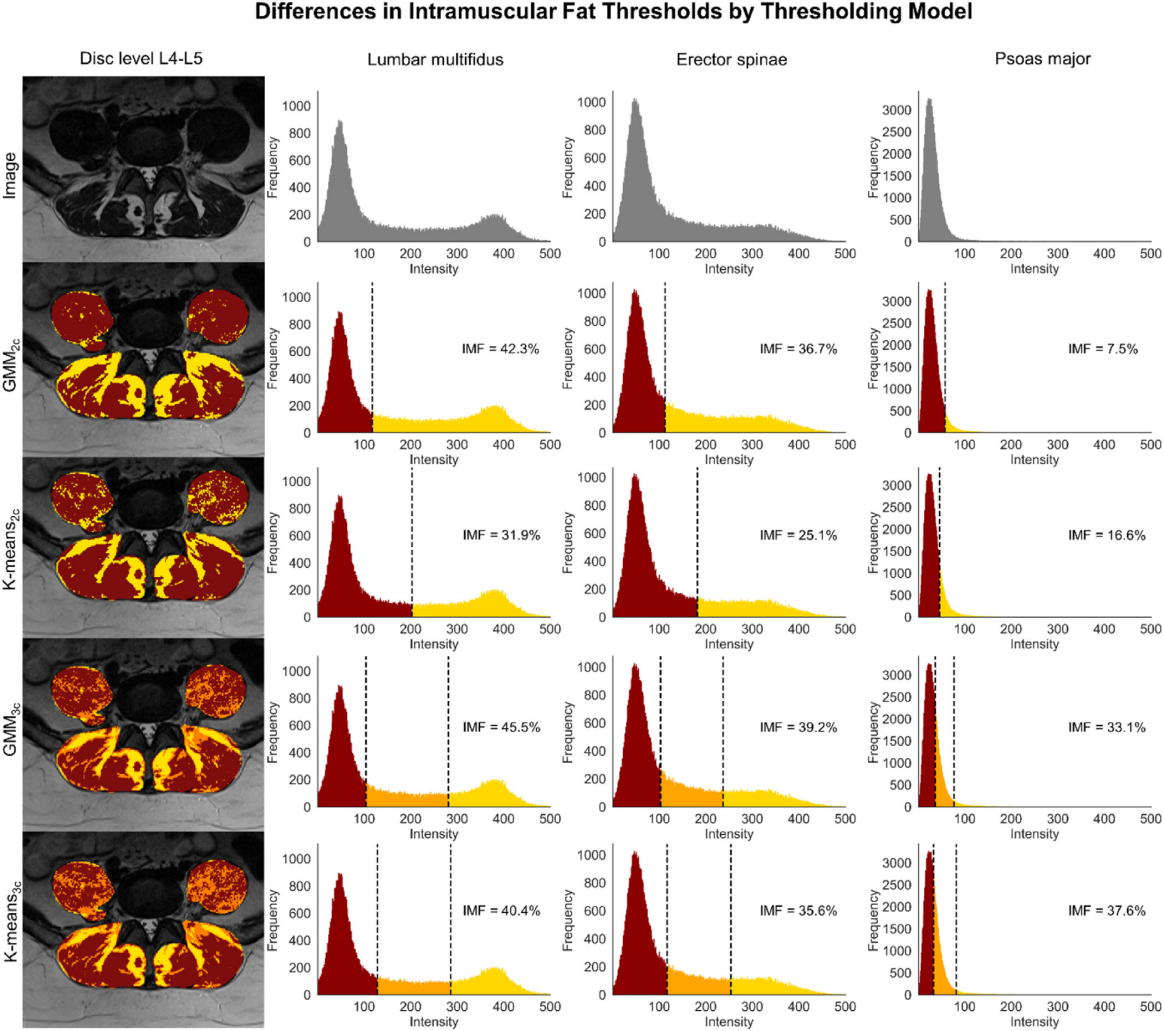
Intramuscular muscle and fat masks, histograms and thresholds for each thresholding model for the lumbar multifidus, erector spinae, and psoas major. First row of images represents the original image at disc level L4–L5, with corresponding histograms for the total region of interest. Red, muscle; orange, undefined area; yellow, fat. The percentage of intramuscular fat is shown at each histogram. Images are from a single representative participant.
(d) Field-of-view. We included the muscle measures from all axial slices from 30 participants with a field-of-view between the mid-vertebral level of L4 and upper endplate of S1. Ornowski et al. [31] averaged the muscle measures from 11 participants using 2 axial slices centered at each lumbar disc level (L1–L5). As the magnitude of lumbar paraspinal IMF is more profound at the lower lumbar region [13], we compared the thresholding models using a region with higher paraspinal IMF (∼29.5% vs. ∼19.5%). As the thresholding performance appears to be dependent on the magnitude of IMF [35], this might also partially explain the differences in outcomes. More research is needed to investigate the thresholding performance across differences in field-of-view and people with varying levels of IMF [36].

Our results contrast with some conclusions drawn by Ornowski et al. [31]. They found that the thresholding models overestimated IMF levels for the multifidus and erector spinae, while our study found an underestimation of IMF with respect to Dixon MRI. Second, Ornowski et al. [31]. concluded that the accuracy of IMF measures is more dependent on which muscle is analyzed than which thresholding approach is used, while we also report significant differences between the thresholding approaches. Despite variations in the sample characteristics, differences between the studies might be explained by:

(a) The number of components. We reached our conclusions by comparing the thresholding models using equivalent number of components. Ornowski et al. [31] used a three-component approach for K-means and two-component approach for the GMM. As our results suggest, the number of components used influences the amount of IMF calculated by the thresholding models.
(b) The initialization procedure. Thresholding models are strongly dependent on the initialization of its parameters [26]. We chose to initialize our models as recommended for optimal thresholding performance, and we initialized the models 50 times to account for the stochasticity in the initialization of the model parameters [32,33]. No specific information about modelling choices (eg initialization methods, number of initializations, or options to constrain the covariance of the GMM) was provided by Ornowski et al. [31]. Consequently, a direct comparison between these studies cannot be made. We encourage researchers to provide detailed information regarding the model parameters used when reporting IMF, as these parameters may influence the assessment of IMF in people with and without LBP.
(c) Vendor, image bit-depth, and intensity correction. Ornowski et al. [31] utilized an MRI-scanner from general electric (GE medical systems) and corrected the T_2_-weighted images using a contrast limited adaptive histogram equalization. Additionally, they converted the T_2_-weighted images to 8-bits, providing grey-scale values for muscle and IMF ranging from 0 to 255. In our study, we utilized a Siemens (Magnetom Vida) scanner with the original bit-depth (16-bits) for the T_2_-weighted images and no intensity correction, providing grey-scale values for muscle and IMF ranging from 0 to 500 (see Fig. 4). Consequently, variability in imaging resolution, sites, and vendors leads to varying signal intensity distributions which likely affects thresholding performance [34], [42]. We are currently working to develop a generic acquisition protocol to standardize the assessment of whole-body IMF across MR manufacturers, similar to recent efforts in the spinal cord MRI field [34]. A consistent and consensus-driven measurement approach should be used in future research to increase methodological consistency and improve the comparison of findings between studies [30]. The programming code used for this study will be freely available (https://github.com/MuscleMap/MuscleMap).

In our study, the three-component thresholding approach improved the accuracy and reliability for quantifying lumbar multifidus and erector spinae IMF. This can be explained by (what we will refer to as) the ‘undefined zone’ (see Fig. 4). The ‘undefined zone’ is a region with uncertain probabilistic membership of either muscle or IMF, partially due to an increase in intramyocellular lipid droplets, which are not visually identified as clear fat depots due to partial volume effects [28]. The consequence of tissue mixing is a voxel intensity histogram with an intermediate zone where the proportions of IMF are likely to be distributed over a wide range of voxel intensities (see Fig. 4) [37]. As such, varying signal intensity distributions can vary both between and even within subjects (ie between slices) [23,34]. Compared to a two-component model, a three-component model (in particular GMM_3C_) potentially provides a more accurate partial volume modelling of IMF (ie capturing the IMF proportions more accurately across the entire fat spectrum) or better model fitting to the varying intensity distributions of IMF [25,38].

The ‘undefined zone’ could additionally be explained by alternative non-contractile tissue infiltrates, such as fibrosis and inflammation, with intermediate voxel intensities falling between the muscle and IMF spectrum [39]. In fact, Shahidi et al. [39] showed the total area occupied by dense and loose collagen (26.1%) was larger than IMF (11.7%). But, this cannot yet be directly quantified with current models [16]. Conventional MRI using three-component thresholding models could, however, have potential benefit for detecting and characterizing multiple tissues (eg muscle, fat, and fibrosis), as shown to be the case in brain MRI [40]. However, more research is needed to investigate the applicability and validity of thresholding techniques for classifying multiple tissue types, as Dixon MRI cannot be used as a reference for classifying fibrosis. Based on our findings, we recommend the use of GMM over K-means and three-component over two-component modelling for the quantification of lumbar multifidus and erector spinae IMF.

Poor performance was observed for the psoas major across all thresholding models, similar to what was shown in 2 previous studies [18,31]. This poor performance can be explained by the low magnitude of IMF resulting in a unimodal instead of multimodal histogram. As such, multicomponent thresholding models are likely to overfit the variance of the intensity distribution in lean muscles, leading to a misclassification for and characterization of muscle tissue as IMF (see Fig. 4). Another explanation for poor IMF quantification in the psoas major, could be that this muscle is located further away from the spinal coil, leading to reduced signal [41]. Correcting for image intensity inhomogeneity could improve the quantification of IMF in the psoas major [41]. More research investigating the applicability of thresholding models in lean muscles and correcting for signal inhomogeneity for the quantification of psoas major IMF is warranted. Given the current state of evidence, we recommend the use of Dixon MRI over T_2_-weighted MRI for the quantification of psoas major IMF.

### Limitations

This study has some limitations. First, we used a relatively small sample to investigate the performance of thresholding models. We chose a sample of 30 people with LBP, as an optimal cut-off between what was minimally recommended for reliability studies [29] and the time-consuming burden of manual segmentation. Next, imaging was not specifically performed for research, and we did not have access to other clinical measures including LBP status (ie duration, intensity, etc.) or other demographic variables such as body mass index (BMI). Not being able to fully characterize the sample of LBP participants may reduce the generalizability of our findings to other datasets with different clinical characteristics. Future studies are needed to investigate the performance of thresholding models using larger, heterogenous clinical samples. Lastly, both sequences (T_2_-weighted and Dixon) were not coregistered. We believe that this concern is mitigated by segmenting the muscles at consistent lumbar levels across participants, ensuring the segmentations were matched for volume.

## Conclusion

We found the performance of automated thresholding models is strongly dependent on the choice of algorithm, number of components, and muscle assessed. Overall, GMM performed better than K-means and three-component models better than two-component models for the quantification of lumbar multifidus and erector spinae IMF. In particular, the three-component GMM thresholding model (GMM_3C_) performed best with good accuracy and reliability with respect to Dixon MRI. None of the investigated thresholding models adequately quantified psoas major IMF. Future research is needed to investigate the performance of thresholding models in a more heterogeneous clinical dataset and across different sites and vendors.

## Author contributions

EOW and KAW designed the study. PP generated the MR images. EOW manually segmented the paraspinal muscles. EOW, KAW, JME, MWC, AP, ADI analysed and interpreted the results. EOW, KAW, JME and MWC prepared all the figures. All authors contributed to various drafts of the manuscript and approved the final version.

## Additional information

The authors certify that they have no affiliations with or financial involvement in any organization or entity with a direct financial interest in the subject matter or materials discussed in the article.

## Data availability

The de-identified datasets used in this study are available from the corresponding author upon reasonable request.

## Financial disclosure

The authors certify that they have no affiliations with or financial involvement in any organization or entity with a direct financial interest in the subject matter or materials discussed in the article.

## Declaration of Competing Interest

One or more of the authors declare financial or professional relationships on ICMJE-NASSJ disclosure forms.

## Appendix Supplementary materials

Image, application 1
