# Supplementary material for: Quantifying lumbar paraspinal intramuscular fat: Accuracy and reliability of automated thresholding models
Source: N Am Spine Soc J. 2024 Jan 24;17:100313. doi: 10.1016/j.xnsj.2024.100313 (PMC10869289; doi:10.1016/j.xnsj.2024.100313)
Supplement: Supplementary file 1 [file mmc1.docx]

**SUPPLEMENTARY FILE**

Algorithm 1: K-means clustering

The main objective of K-means is to minimize the sum of squared errors between the voxel values and muscle and fat components[1], given by the following objective function

$$\sum_{k=1}^{k} \sum_{i=1}^{n} || x_{i}- \mu_{k}||^{2}$$

with *k* components, $x_{i}$ is the intensity value of one voxel, and$\mu_{k}$ is the mean intensity centroid value of the corresponding tissue component.

The muscle and fat component centroids are initialized at the first iteration and assigned to a component with the lowest variance with respect to the component centroid. The new mean intensity value $\mu_{k}^{new},$given a hard component assignment $\left\{ c^{i} = k \right\}$ of the *kth* component, is updated by averaging all voxel intensity values from the previous iteration:

$$\mu_{k}^{\mathrm{new}}= \frac{\sum_{i=1}^{m} \left\{ c^{i} = k \right\} x_{i}}{\sum_{i=1}^{m} \left\{ c^{i} = k \right\}}$$

The algorithm continues until the change in the cost-function converges, or until the cut-off point for maximal iterations have been reached.[1]

Algorithm 2: Gaussian Mixture Modelling

GMM involves assigning voxel-specific probabilities to latent underlying gaussian density functions, characterizing an overall distribution for each tissue component.[2] The Gaussian mixture is a set of distribution probabilities representing tissue components with independent parameters given by the general formula:

$$\sum_{k=1}^{k} \sum_{i=1}^{n} \pi_{k} \mathcal{N}\left( x_{i} | \emptyset_{i} \right)$$

Here, each component function$\mathcal{N}\left( x_{i} | \emptyset_{i} \right)$ is the *k*th gaussian distribution with parameter $\emptyset$ containing a mean vector μ_k_ and covariance matrix  Σ_k_. The prior probability (or weight) π_k_ is independent of observation $x_{i}$, and represents the proportion of the voxel value belonging to muscle or fat, normalized by the total population. The Expectation-Maximization (EM) algorithm is commonly used to obtain and update the maximum likelihood estimates from the parameters of a mixture model.[3] The EM algorithm alternates between (a) estimating the posterior probability of a voxel value belonging to muscle and fat, assuming that the parameters ($\mu$*_k_*_,_  $\Sigma$_k_) are known and (b) updating the estimates of the parameters by fixing the posterior probabilities of the muscle and fat components.[3] The means, covariances and mixing coefficients (prior probability) are initialized at the first step. In the second step, the posterior probability $\gamma\left( Z_{ki} \right)$ is calculated using the initialized parameters. In the third step, the parameters ($\mu_{k}^{new}$, $\Sigma_{k}^{new}$, $\pi_{k}^{new}$) are updated given the posterior probability $\gamma\left( Z_{ki} \right)$.[3] The algorithm continues until the change in the maximum likelihood converges, or until the cut-off point for maximal iterations is reached.

$$\gamma\left( Z_{ki} \right)=\frac{\pi_{k}\mathcal{N}\left( x_{i} | \emptyset_{i} \right)}{\sum_{k=1}^{k} \pi_{k}\mathcal{N}\left( x_{i} | \emptyset_{i} \right)}$$

The new mean $\mu_{k}^{new}$ for each component is updated by averaging the sum over the posterior probability of the *kth* component $\sum_{i=1}^{N} \gamma(Z_{ki})$multiplied by each voxel value $x_{i}$.

$$\mu_{k}^{new}= \frac{\sum_{i=1}^{N} \gamma(Z_{ki}) x_{i}}{\sum_{i=1}^{N} \gamma(Z_{ki})}$$

The new covariance matrix for each component is updated by calculating the sum over the posterior probability$\sum_{i=1}^{n} \gamma(Z_{ki})$multiplied by the variance$(x_{i}- \mu_{k}^{new}) (x_{i}- \mu_{k}^{new})^{T}$. The size of the covariance matrix (rows x columns) is dependent on how the covariance type is initialized (e.g. ‘full’, ‘diagonal’, ‘spherical’, ‘tied’).

$$\Sigma_{k}^{new}= \frac{\sum_{i=1}^{n} \gamma(Z_{ki}) (x_{i}- \mu_{k}^{new}) (x_{i}- \mu_{k}^{new})^{T}}{\sum_{i=1}^{n} \gamma(Z_{ki})}$$

The new weight $\pi_{k}^{new}$ was updated by normalizing all voxels belonging to the *kth* component over the total amount of voxel values.

$$\pi_{k}^{\mathrm{new}}=\frac{N_{k membership}}{N_{total membership}}$$

Once the EM-maximization process is converged, all voxels ($\forall_{i})$ are assigned to a *kth* ($\forall_{k})$ component with the highest posterior probability $arg max \gamma\left( Z_{nk} \right)$ for the hard component assignment.

$$C^{i}= arg max \gamma\left( Z_{ki} \right) , \forall_{k}, \forall_{i}$$

**SUPPLEMENTARY TABLE 1.** Mean (SD) percentage of intramuscular fat for the four thresholding models and the ground truth (Dixon MRI).

| **Muscle** | **GMM_2C_** | **GMM_3C_** | **K-means_2C_** | **K-means_3C_** | **Dixon MRI** |
| --- | --- | --- | --- | --- | --- |
| Lumbar multifidus (left) | 30.0 (8.4) | 32.2 (9.2) | 20.8 (7.6) | 30.1 (9.4) | 36.3 (11.4) |
| Lumbar multifidus (right) | 30.1 (7.8) | 32.3 (8.5) | 20.7 (7.2) | 30.1 (8.7) | 37.4 (11.1) |
| Erector spinae (left) | 29.0 (9.1) | 33.4 (10.4) | 21.2 (8.5) | 32.7 (10.4) | 38.5 (12.4) |
| Erector spinae (right) | 31.1 (8.1) | 36.5 (8.9) | 22.1 (7.9) | 35.7 (8.8) | 38.0 (12.1) |
| Psoas major (left) | 9.3 (2.8) | 35.0 (6.7) | 20.7 (7.2) | 41.8 (6.4) | 12.9 (2.6) |
| Psoas major (right) | 8.5 (2.8) | 32.6 (5.4) | 18.9 (6.7) | 39.2 (5.6) | 13.3 (2.4) |

GMM: Gaussian Mixture Model; 2C and 3C refer to algorithms with two or three components, respectively.

**REFERENCES**

[1] Gray C, MacGillivray TJ, Eeley C, Stephens NA, Beggs I, Fearon KC, et al. Magnetic resonance imaging with k-means clustering objectively measures whole muscle volume compartments in sarcopenia/cancer cachexia 2011;30:106–11.

[2] Kim N, Heo M, Fleysher R, Branch CA, Lipton ML. A Gaussian Mixture Model Approach for Estimating and Comparing the Shapes of Distributions of Neuroimaging Data: Diffusion-Measured Aging Effects in Brain White Matter. Front Public Heal 2014;2:32. https://doi.org/10.3389/FPUBH.2014.00032.

[3] Ng SK, Krishnan T, McLachlan GJ. The EM Algorithm. Handb Comput Stat 2012:139–72. https://doi.org/10.1007/978-3-642-21551-3_6.
